# Supplementary material for: Snail-inspired water-enhanced soft sliding suction for climbing robots
Source: Nat Commun. 2024 May 13;15:4038. doi: 10.1038/s41467-024-48293-2 (PMC11091155; doi:10.1038/s41467-024-48293-2)
Supplement: Supplementary file 1 — Supplementary Information [file 41467_2024_48293_MOESM1_ESM.pdf]

Supplementary Information for

Snail-inspired Water-enhanced Soft Sliding Suction for Climbing Robots

Authors

Tianqi Yue[1], Hermes Bloomfield-Gadêlha[1], and Jonathan Rossiter[1*]

[1] Department of Engineering Mathematics and Bristol Robotics Laboratory, University of Bristol, Bristol, UK.

This file contains:

Supplementary Sections S1 to 9

Supplementary Figs. 1 to 8

Supplementary Table 1

Supplementary References

S1 PBP working principle on improving the silicone wettability

PBP is a hydrophilic pendant chain which can stably anchor to the silicone polymer and generate attractive forces to water molecules[1]. The working principle is shown in Fig.S1. PBP makes the bulk of the silicone hydrophilic, in contrast to other modification methods such as plasma treatment which only improves the silicone surface wettability.

S2 Design and structural details of SSR

The design of SSR begins with the suction cup. The fundamental structure of the suction cup, as we describe in the main text, is a double-layer structure composed of a top PU reinforcement layer and a bottom PBP-silicone pad. The diameter of the suction cup is selected as 50 mm which is a suitable size for practical demonstrations. The key geometrical parameter of the suction cup design is the slope angle, as shown in Supplementary Fig. 2. We tested three slope angles with 10°, 20° and 30° and no difference was observed between the sliding suction performance of three suction cups, while the 10° suction cup generates a slightly larger maximum suction force than the other two. It can be explained by Equation (1) in the main text, $F_{\mathrm{pull}} = (p_{\mathrm{atm}} - p_{\mathrm{in}})A - F_{\mathrm{bend}} + mg$. Larger slope angle makes the suction cup generate larger bending force against the substrate, therefore the maximum suction force is accordingly reduced. Considering the application scenario of the current design is sliding on flat and smooth surfaces, 10° is considered the optimal slope angle.

The second important design is to control the attachment and detachment of the suction cup. The action of the suction cup attaching to the substrate builds a new seal and detachment from the substrate requires breaking of this seal. A mechanism should be therefore needed to place and lift the suction cup rim to build and break the seal respectively. We use a snapping membrane to achieve this, adopting a similar mechanism as proposed in our previous work[2,3] and shown in Supplementary Fig. 3. An air chamber is composed of a rigid upper lid (6 in Supplementary Fig. 4) and a soft snapping membrane (5 in Supplementary Fig. 4). On the upper lid there is a thin through-hole with 0.5 mm diameter. The snapping membrane is monostable: when the air chamber holds a negative pressure (vacuumed by the pump, ~<20 kPa), the toroidal-shaped snapping membrane snaps backward; therefore, this backward contraction lifts the suction cup rim (i.e., detaching). Due to the mono-stability, when the air pump stops vacuuming, air leaks into the chamber through the hole on the upper lid and the snapping membrane

snaps forward to place down the suction cup rim (i.e., attaching). This design allows the SSR to use just one tiny air pump to control the states of the suction cup, avoiding the need for a complex circuit.

The third critical design is the driving system for achieving sliding movement. As shown in Fig. 2 in the main text, we use two wheels to generate sliding movement. Although the sliding suction cup generates a very small kinetic friction force (Fig. 1E and F) during the sliding, we still require the wheels to generate sufficient static friction force, depending on the situation, e.g., to overcome the gravity during vertical climbing. Based on the analysis of the perpendicular pulling payload force on the kinetic friction in the main text, we know that pulling the suction cup does not significantly increase the kinetic friction. However, pushing the wheels onto the substrate, of which the tyre is made from hydrophobic silicone (Dragon Skin 10 NV), significantly increases their static friction and grip. Therefore, we can utilise the antagonistic force between the suction cup and wheels to increase the wheels' static friction. We use a circular array by six compressive springs (7 in Supplementary Fig. 4) to generate the antagonistic force between the suction cup and wheels. The selection of the springs' parameter is determined considering appropriate force and size. After that, DC motors and gears were designed to drive the movement.
The final design is the control system. We use a Wemos D1 mini board to receive controlling signals from a PS4 game controller, and two L293D motor drivers to respectively control the two DC motors and two pumps.

The detailed structure of SSR is shown in the exploded diagram in Supplementary Fig. 4. Explanations of each component are provided in Supplementary Table 1.

S3 Fabrication method of reinforcement layer and PBP-silicone bottom pad

The reinforcement layer is made by following steps. First, prepare the molds by 3D printing and spray mold release. Second, weigh part A and B of liquid PU rubber by the mass ratio of 1:1, then mix it well. Third, degas the liquid mixture of PU rubber in the vacuum chamber for 2 minutes, then quickly pour it into the molds. Fourth, bake the molds in oven at 40 ^{o}C for 1 hour. Fifth, disassemble the molds to obtain the cured reinforcement layer.

The PBP-silicone bottom pad is made by following steps. First, prepare the molds by FDM-3D-printing, and polish the inner surface using sandpaper with grit larger than 1600, until the surface is smooth.

Second, weigh part A and B of liquid silicone by the mass ratio of 1:1, with total mass *M* g. Third, weigh 0.2*M* g PBP, then mix liquid silicone and PBP thoroughly. Fourth, pour the liquid mixture into the bottom mold. Fifth, degas the liquid mixture in the vacuum chamber for 10 minutes, making sure no bubbles are visible. Sixth, assemble the molds carefully. Seventh, bake the molds in oven at 40 ^{o}C for 4 hours. Eighth, disassemble the molds to obtain the cured PBP-silicone bottom pad.

S4 Fabrications and assembling methods of SSR

We first prepared all components as shown in Supplementary Fig. 5, then assembled the SSR via following steps as shown in Supplementary Fig. 6:

- Step 1: We soldered electric components, assembled them onto the chassis of the SSR and sealed electric components with silicone rubber (Ecoflex 00-30). We assembled the upper lid and snapping membrane with instant glue (Loctite Precision Max).
- Step 2: We localised electric components onto supporting plates on the chassis, and assembled gears and wheels onto the chassis. We assembled the suction cup (composed of the PU reinforcement layer, PBP-silicone pad, SA foam and tubes) beneath the snapping chamber via soft glue (Sil-poxy, Smooth-on).
- Step 3: We assembled the suction unit with the chassis via instant glue.
- Step 4: We connected all tubes to pumps and assembled the water tank to the back of the SSR."

S5 Detailed force analysis and calculations

S5.1 Sliding ability

The "sliding ability" we refer to is the ability of the SSR to slide on the substrate. Since gravity acting on the SSR and the pulling force may add force components at the suction interface, we calculate the sliding ability in the simplest condition. No external pulling force is exerted on the SSR and the SSR is upside-down adhering to the PMMA ceiling (no tangential gravity component).

The equivalent pulling force $F_{\text{pull\_equ}}$ is

$$F_{\text{pull\_equ}} = 6F_{\text{spring}} + mg = 6kd_{\text{spring}} + mg,$$

where k = 0.15 N/mm is the spring constant of each spring, d_{spring} = 3.5 mm is the compression distance and mg= 0.94 N is the gravitational force on the SSR. The kinetic friction force to overcome is

$$f_{\text{kinetic}} = \hat{\mu}_{\text{wat\_suc}} F_{\text{bend}}\left(F_{\text{pull\_equ}}\right) = 0.59 \text{ N}.$$

In the meantime, the maximum static friction force that two wheels can supply is

$$f_{\text{tyre}} = \hat{\mu}_{\text{wat\_tyr}} \cdot 6F_{\text{spring}} = \hat{\mu}_{\text{wat\_tyr}} \cdot 6kd_{\text{spring}} = 17.51 \text{ N.}$$

The driving force of the SSR comes from two DC motors. When supplied with 5 V, the output motor torque is $\tau_{\text{motor}} = 37.2$ mN·m. The torque is transited through the gears with gear ratio $\eta = 11/28$ to two wheels. Therefore, the total maximal driving force on the wheels is

$$F_{\text{drive}} = \frac{2 \cdot 0.8 \cdot \tau_{\text{motor}}}{\eta r_{\text{wheel}}} = 16.834 \text{ N,}$$

where 0.8 is the estimated torque transition efficiency in gears, $r_{\text{wheel}} = 9$ mm is the radius of the wheel including tyre. Based on the former calculations, we know that $f_{\text{kinetic}} < F_{\text{drive}} < f_{\text{tyre}}$, indicating that the kinetic friction force at the suction interface can be easily overcome by the driving force, while they are both less than the static friction force on the tyres (no tyre slip). Therefore, the SSR can easily slide upside-down on the PMMA ceiling.

S5.2 Climbing ability

Here the "climbing ability" we refer to is the ability of the SSR to vertically climb upward on the wall, which is the most difficult condition since the total gravitational force from the mass of the SSR acts in direct opposition to upward climbing. The equivalent pulling force applied on the SSR becomes

$$F_{\text{pull\_equ}} = 6F_{\text{spring}}.$$

The total tangential force that needs to be overcome is the sum of the kinetic friction force at the suction interface and the gravity,

$$f_{\text{kinetic}} + mg = \hat{\mu}_{\text{wat\_suc}} F_{\text{bend}}\left(F_{\text{pull\_equ}}\right) = 1.52 \text{ N.}$$

Again, we know that $(f_{\text{kinetic}} + mg) < F_{\text{drive}} < f_{\text{tyre}}$, therefore SSR can easily climb upward on the PMMA wall.

S5.3 Payload ability

Here the "payload ability" is the total pulling force that the SSR can maintain suction against when sliding upside-down on the ceiling (the simplest case). We consider a payload mass m_{load} applied perpendicular to the SSR when it is sliding upside-down on the PMMA sheet. The equivalent pulling force becomes

$$F_{\text{pull\_equ}} = 6F_{\text{spring}} + mg + m_{\text{load}}g.$$

Therefore, increasing the payload mass causes f_{kinetic} to increase. Let the kinetic friction force equal the driving force when the two motors are operating at their rated power,

$$f_{\mathrm{kinetic}} = \hat{\mu}_{\mathrm{wat\_suc}} F_{\mathrm{bend}}\left(F_{\mathrm{pull\_equ}}\right) = F_{\mathrm{drive}} = 16.83 \text{ N}.$$

Solving the equation we obtain $m_{\mathrm{load\_max}} = 46.67$ kg (an equivalent payload force of > 460 N), the maximum mass that an ideal SSR can carry. In experiments, we measured a maximum stationary suction force for the SSR of 50.3 N (5.03 kg). This also defines the practical upper bound for loaded sliding using the fabricated suction cup in the SSR. We limited our loaded sliding tests to 1 kg to avoid damaging the robot, which is mainly 3D-printed, and the suction cup, which is non-optimised. The calculated result (above) shows the extraordinary payload potential of SSR, even though it is only driven by two small 5 V DC motors.

S6 Measurement of the suction cup bending force F_{bend} and maximum stationary suction force

The force characterization method of suction cups has been well reported[4,5]. We mounted the SSR on the linear stage slider and placed a PMMA sheet below it. The PMMA sheet was laser cut with a tiny through-hole (~0.5 mm diameter) to let air freely flow in and out. A load cell was connected beneath the PMMA sheet to measure the contact force. We covered the PMMA sheet with a film of 5% w/w detergent solution to eliminate friction, then controlled the linear stage slider to slowly move the SSR down to contact with the PMMA sheet. Once the force became non-zero (indicating the contact occurred), the position of SSR was recorded. The linear stage continued to move down for a further 2 mm, then stopped. 2 mm is the depth of the suction disc, so SSR was deemed to be just flattened. The force recorded by the load cell at this moment is the measured F_{bend} when $F_{\mathrm{pull}} = 0$.

To measure the maximum stationary suction force, we first let the suction cup statically adhere to the PMMA substrate (without a through-hole this time), then controlled the linear stage slider to move the suction cup away from the substrate. The measured breaking-off force is the maximum stationary suction force.

S7 Calculation of the energy and water consumption

The power consumption can be calculated by following method. We assume that SSR slides upside-down on the PMMA sheet, carrying the 1 kg mass. The power consumption comes from: two DC motors driving power (2×58 mA $\times 5$ V $= 0.58$ W); the MCU board operating power including

Bluetooth (~ 170 mA $\times$ 3.3 V $= 0.561$ W); the water pump intermittent (~ 0.3 of the constant pumping) supplying power (0.3×160 mA $\times$ 3 V $= 0.48$ W); the voltage booster power conversion efficiency (95%). Therefore, the total maximum power consumption during sliding is $(0.58 + 0.561 + 0.48)/0.95 = 1.7$ W. For our experiments including rotational, translational and loaded sliding, the power consumption is even less since all the electronics were not working at their rated power. The robot is able to statically adhere to the substrate with all the electronics shut down, therefore the power consumption is 0 W.

The water film thickness ≈ 0.05 mm and the inner volume of the hemispheric portable tank is 16.7 cm3. Sliding of the SSR leaves a water film with 54 mm width, therefore the portable water tank can provide $16.7/(5 \times 10^{-3} \times 5.4) \approx 619$ cm sliding.

S8 Control the SSR via a wireless game controller

Pushing the “Triangle” button on the controller causes the air pump to work for 2 seconds and invert the suction cup ready for attaching. Pushing the “Cross” button causes the water pump to secrete water to wet the suction interface. The “Left” or “Right” buttons cause the robot to rotate clockwise or anticlockwise. The “Up” or “Down” buttons cause the robot to translate forward or backward. Translational movement is achieved by rotating the wheels in the same direction, while rotation is achieved by rotating the two wheels in opposite directions.

S9 Discussion of the pump-assisted design for sliding on rough surfaces

To determine the influence of surface roughness on sliding suction performance, we conducted a series of experiments on rough surfaces. Three rough surfaces were prepared by engraving flat PMMA sheets. We use a FusionEdge (EpilogLaser) laser cutter to engrave parallel lines (0.5 mm distance) with different depths. From surface 1 (denoted as S1, smoothest) to surface 3 (denoted as S3, roughest), we set the engraving laser parameters as 100 % speed, 5 / 10 / 15 % power and 5000 Hz. The engraved surfaces are shown in Supplementary Fig. 7A. To measure the pressure inside the suction region, we drilled a thorough-hole (1 mm diameter) on the bottom centre of the suction cup, and connected the hole with thin silicone tubes to a pressure sensor (SSCDRRV015PDAA5, Honeywell). Data was read by an Arduino Nano board. Experiments were conducted via following steps: First, we wet the rough surface with a thin film of water. Second, we manually squeezed the SSR to the surface then released it, from

which the suction is deemed to begin (as the black dashed line shows in Supplementary Fig. 7B). We separate the suction into two states: static and sliding. For static suction, we did not activate the movement of SSR; for the sliding suction, we activate the forward sliding movement parallel to the grooves at the moment of release (black dashed line). According to Supplementary Fig. 7B, surface roughness reduces both the maximum suction pressure and the suction longevity. In addition, the sliding movement does not affect the maximum suction pressure on rough surfaces compared with the static state; however, the suction longevity is reduced by approximately 50 %. During the sliding, new percolation channels between the environment and the suction region are introduced by the relative movement of the two contacting surfaces. We attribute the longevity reduction caused by the sliding movement to this dynamic change in leakage channels. In contrast, the percolation channels of a static suction cup are relatively stable and constant, and their number and form only change by leakage-induced shape recovery of the suction cup. We will not analyse more on the micro contact mechanics[6-8] since this is not the focus of this paper; however, given the experimental results in Supplementary Fig. 7, we can draw the conclusion that the sliding-induced leakage is in the same order of magnitude as the roughness-induced leakage.

However, this does not mean that the sliding suction cannot be applied to rough surfaces. Additional mitigation can be employed to maintain suction; for example, a small vacuum pump can be connected to the suction region to compensate for leakage. This design is similar to traditional wall-climbing robots[9,10], however, the SSR will consume much less energy according to our analysis below. Previous pump-assisted wall-climbing robots require the body to be lifted 1-5 mm (typically 5 mm for commercial products[11]) from the substrate to avoid direct contact and friction; however, this causes severe leakage and accordingly needs a strong vacuuming flow. In contrast, the sliding suction method employs direct body contact with the substrate. The direct contact with a rough surface (e.g., coarse sandpaper with grit >40) leaves a mean gap of ~200 µm (we use half of the granule size of the 40-grit sandpaper for estimation)[12]. According to the leakage model[5], the leakage rate Q of a leaking suction cup can be estimated by

$$Q = \frac{L_y d^3 (p_\mathrm{a} - p_\mathrm{b})}{12 \eta L_x},$$

where L_x and L_y are the width and length of the sealed region of the suction cup, p_a and p_b are the pressure of the outer environment and the internal suction region, d is the gap width (for the pump-

assisted wall-climbing robot, we use $d = 5$ mm; for the pump-assisted sliding suction robot, we use $d = 200$ μm), η is the viscosity of the fluid (for the pump-assisted wall-climbing robot, the fluid is air so $\eta = 5 \times 10^{-5}$ Pa·s; for the pump-assisted sliding suction robot, the fluid is water so $\eta = 1 \times 10^{-3}$ Pa·s). Assuming they both require the same pressure differential to adhere to the substrate (typically $p_\mathrm{a} - p_\mathrm{b} = 2.5{\sim}4.5$ kPa[9,10]) and have the same size (i.e., same L_y/L_x), a simple calculation can derive the ratio of the flow rate:

$$\frac{Q_\mathrm{centrifugal}}{Q_\mathrm{sliding\_suction}} = \frac{d^3_\mathrm{centrifugal}\eta_\mathrm{water}}{d^3_\mathrm{sliding\_suction}\eta_\mathrm{air}} = 3.125 \times 10^5$$

The ratio of the power consumption for maintaining the suction is:

$$\frac{P_\mathrm{centrifugal}}{P_\mathrm{sliding\_suction}} = \frac{Q_\mathrm{centrifugal}d_\mathrm{centrifugal}\rho_\mathrm{air}}{Q_\mathrm{sliding\_suction}d_\mathrm{sliding\_suction}\rho_\mathrm{water}} \approx 1.01 \times 10^4.$$

This indicates that the power consumption on a rough surface (~40 grit) of a pump-assisted sliding suction robot is four orders of magnitude less than a regular pump-assisted wall-climbing robot, demonstrating a significant advance. In addition, we also consider comparing the pump-assisted sliding suction robot with wall-climbing robots based on the Bernoulli effect, which uses a reverse high-speed airflow. A Bernoulli-effect-based climbing robot requires a much higher flow rate to generate the pressure drop than suction-based robots, which consumes significantly more energy[13].

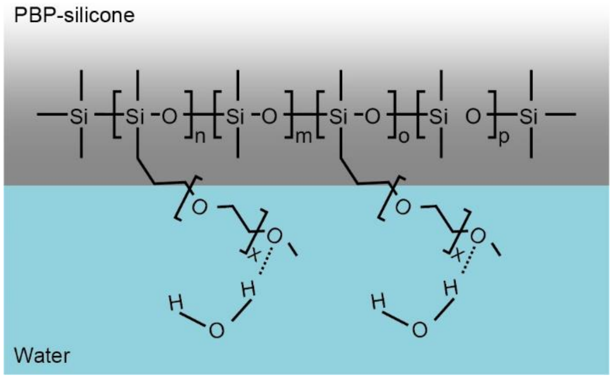

Supplementary Fig. 1 Working principle of PBP for improving silicone hydrophilicity.

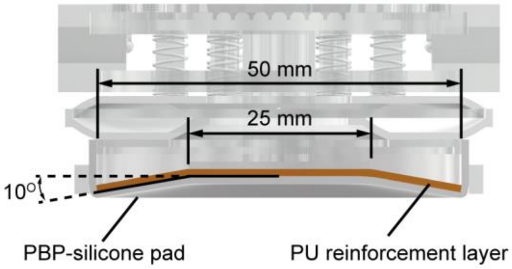

Supplementary Fig. 2. Geometrical dimensions of the suction cup structure.

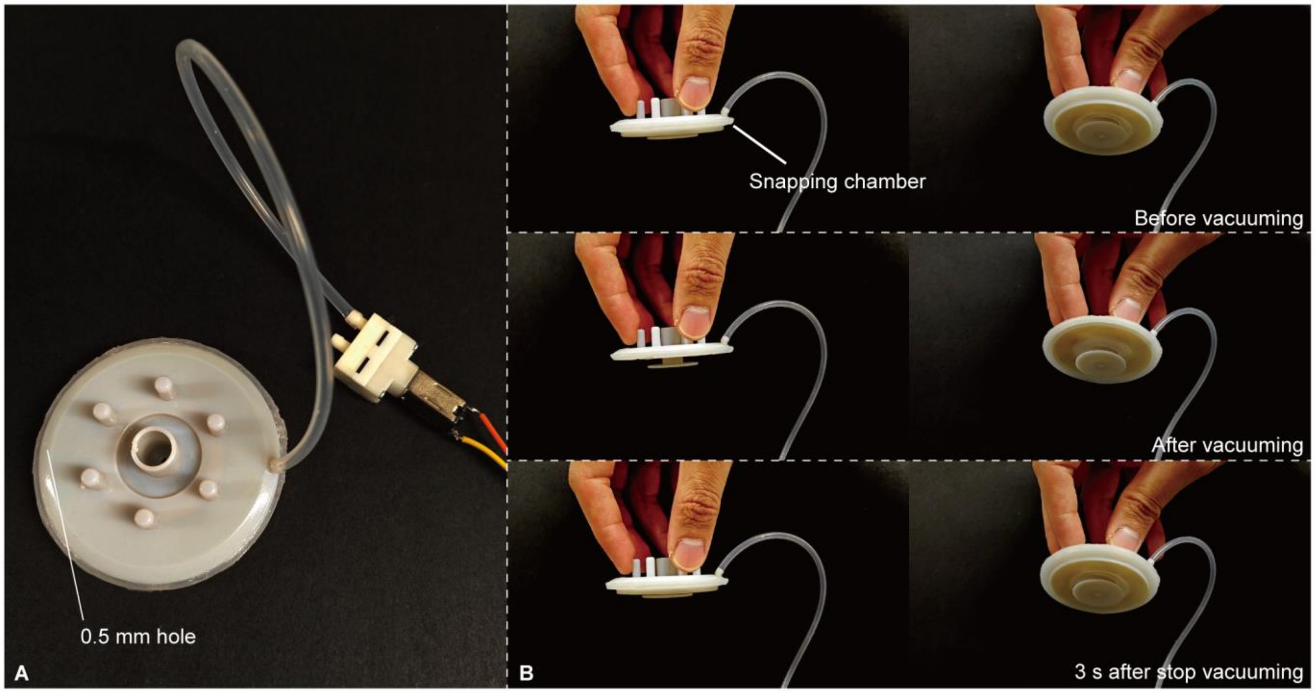

Supplementary Fig. 3. The activation of the snapping chamber via a small diaphragm pump. (A) The pneumatic circuit for activating the snapping chamber. (B) Left: side view during the activation. Right: perspective view.

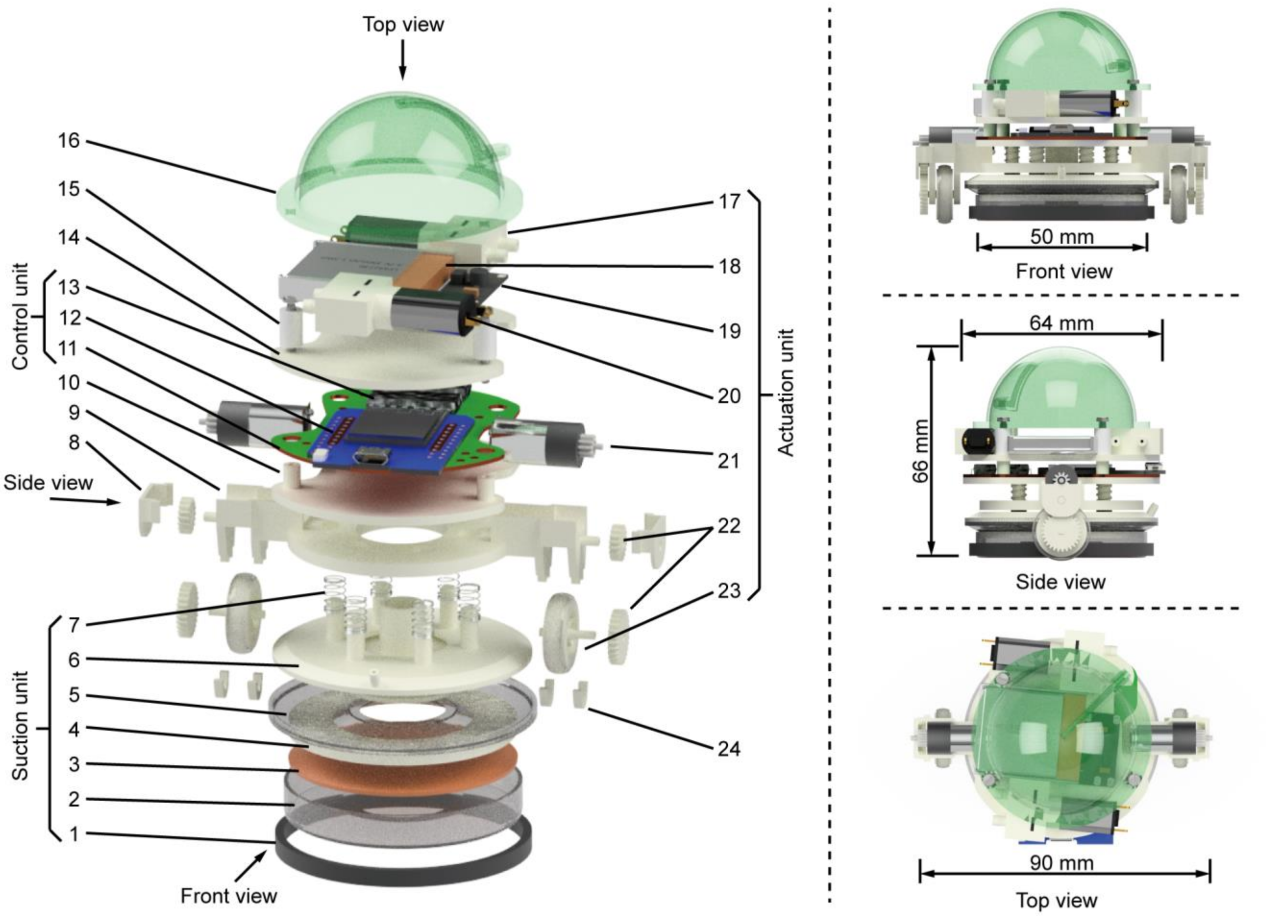

Supplementary Fig. 4. The detailed structure of the SSR. 1~24 components are explained in Supplementary Table 1. Silicone tubes (for air and water transportation) and wires are not shown.

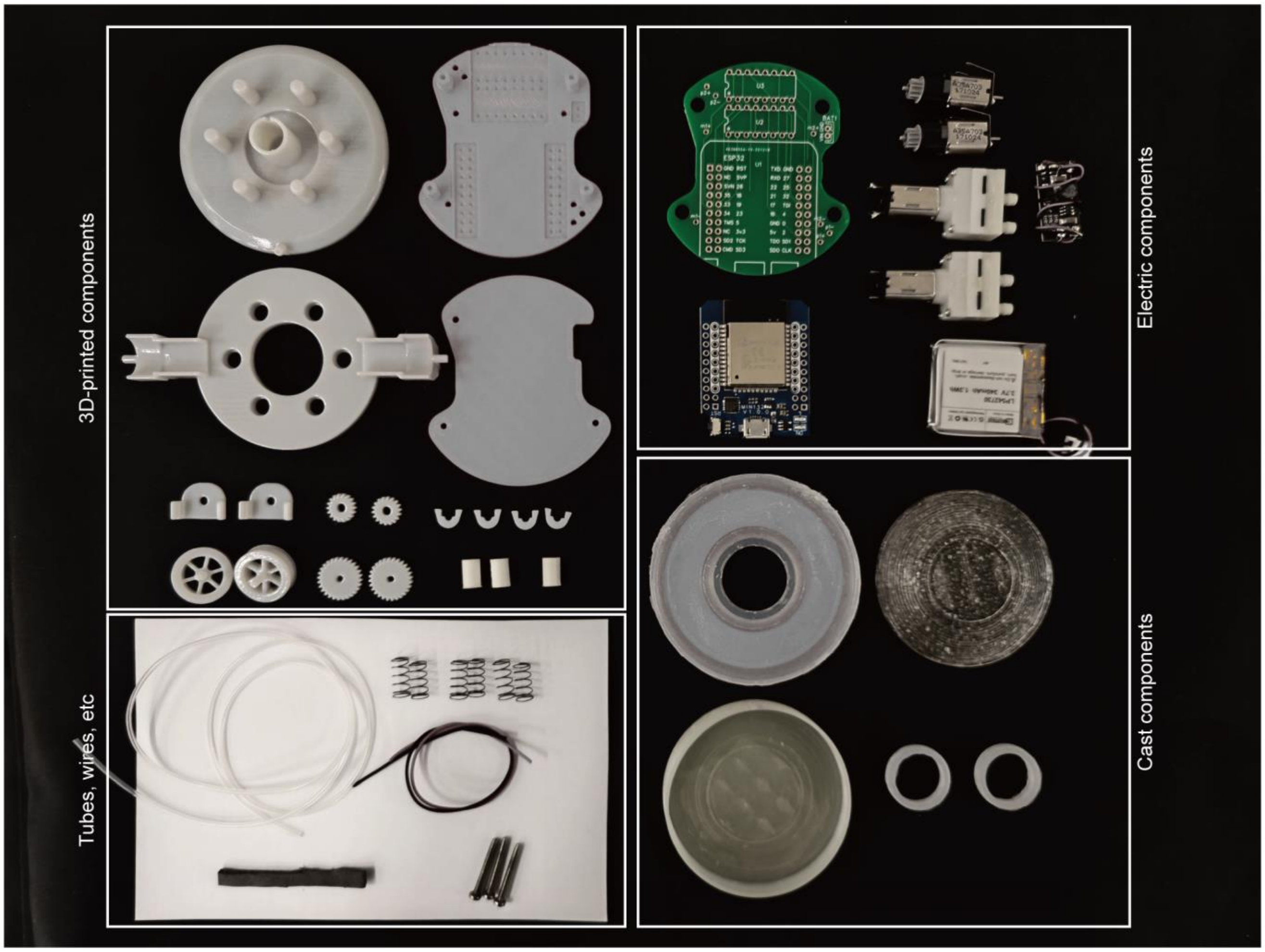

Supplementary Fig. 5. Components for assembling the SSR.

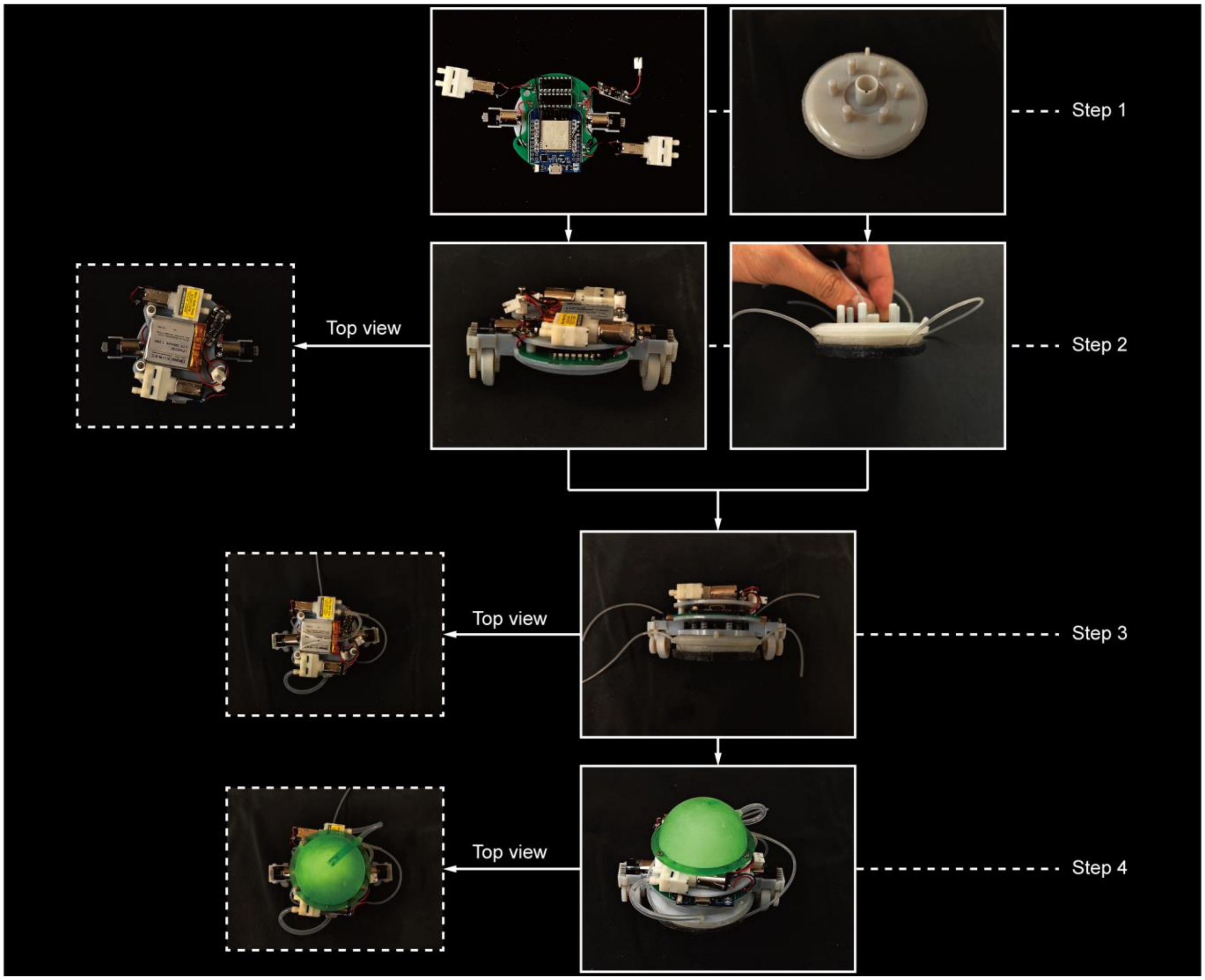

Supplementary Fig. 6. The assembling steps of the SSR.

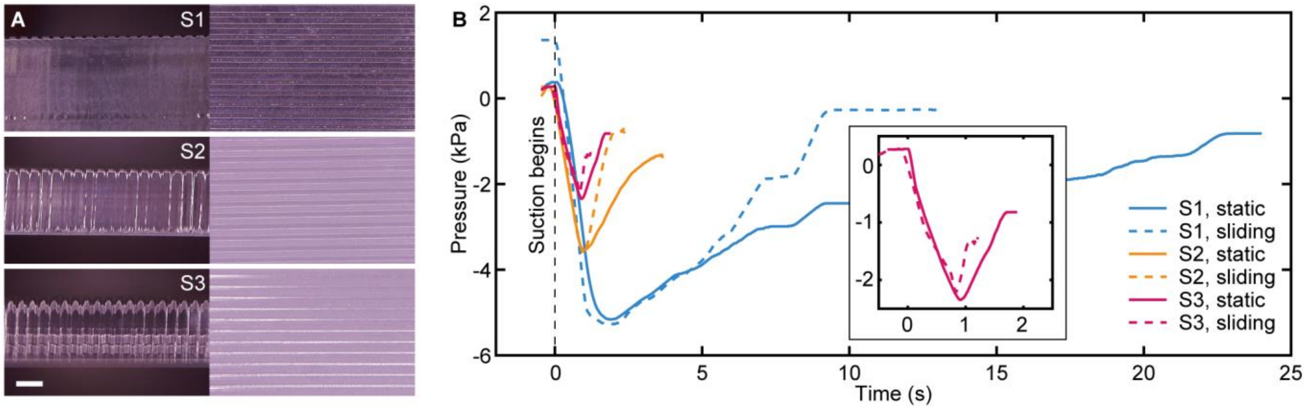

Supplementary Fig. 7. Sliding suction test on rough surfaces. (A) Rough grooved surfaces prepared by laser engraving. Scale bar: 1 mm. (B) Measured suction pressure on rough surfaces in static and sliding suction situations.

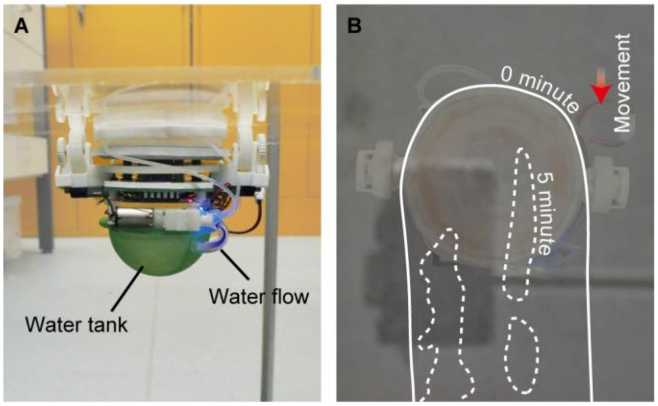

Supplementary Fig. 8. Untethered SSR sliding upside-down on a PMMA sheet. (A) The untethered SSR with a portable water tank on its back. (B) The water mark left on the substrate can evaporate quickly within 5 minutes and with no residue.

Supplementary Table 1. Information of key components and electronics.

No.	Name	Specifications	Function
1	SA foam	Thickness 2 mm	Water reservoir
2	PBP-silicone	Thickness 1 mm, Φ50 mm	Generate capillary force to water
3	PU reinforcement layer	Thickness 1 mm, Φ50 mm	Strengthen the suction cup
4	Constraining plate	Thickness 1 mm, Φ50 mm	Improve the mono-stability of snapping membrane
5	Snapping membrane	Thickness 1 mm	Lift/put down the suction cup rim
6	Upper lid	With a Φ0.5 mm hole	Connecting; leaking for putting down the suction cup rim
7	6 compressive springs	5×0.3×10 mm, 0.15 N/mm	Enhance friction on wheels
8	Supporting piece 1	N/A	Supporting
9	Chassis	N/A	Supporting
10	Supporting plate 1	N/A	Supporting
11	Printed circuit board	N/A	N/A
12	MCU	Wemos d1mini 32	Computing
13	Motor driver	L293D	Control motors
14	Supporting plate 2	N/A	Supporting
15	Supporting piece 2	N/A	Supporting
16	Water tank	3D printed by SLA printer	Reserve water
17	Diaphragm Pump 1	3.7~6 V	Supply water
18	Battery	3.7 V, 340 mAh	Power supply
19	Voltage booster	3.7 V to 5 V	Regulate voltage
20	Diaphragm Pump 2	3.7~6 V	Activate snapping chamber
21	DC motor	2.5~5 V, max 37.2 mN · m	Movement
22	Gears	11:18:28 (top to bottom)	Transmission
23	Wheel with silicone tyre	Φ18 mm	Generate static friction
24	Supporting piece 3	N/A	N/A

Supplementary References

1 Yao, M. & Fang, J. Hydrophilic PEO-PDMS for microfluidic applications. *Journal of Micromechanics and Microengineering* **22**, 025012 (2012). https://doi.org:10.1088/0960-1317/22/2/025012

2 Yue, T., Bloomfield-Gadêlha, H. & Rossiter, J. Shape-Conformable Suction Cups with Controllable Adaptive Suction on Complex Surfaces. *IEEE Robotics and Automation Letters* (2023).

3 Yue, T. *et al.* A contact-triggered adaptive soft suction cup. *IEEE Robotics and Automation Letters* **7**, 3600-3607 (2022).

4 Ge, D. *et al.* Quantitative study on the attachment and detachment of a passive suction cup. *Vacuum* **116**, 13-20 (2015).

5 Tiwari, A. & Persson, B. N. J. Physics of suction cups. *Soft Matter* **15**, 9482-9499 (2019). https://doi.org:10.1039/C9SM01679A

6 Bottiglione, F., Carbone, G. & Mantriota, G. Fluid leakage in seals: An approach based on percolation theory. *Tribology International* **42**, 731-737 (2009).

7 Lorenz, B. & Persson, B. Leak rate of seals: Comparison of theory with experiment. *Europhysics Letters* **86**, 44006 (2009).

8 Persson, B., Albohr, O., Creton, C. & Peveri, V. Contact area between a viscoelastic solid and a hard, randomly rough, substrate. *The Journal of chemical physics* **120**, 8779-8793 (2004).

9 Ecovacs. *Ecovacs Window Cleaning Robot*, <https://www.ecovacs.com/us/winbot-window-cleaning-robot/winbot-w1-pro> (2023).

10 Fang, Y. *et al.* Design and optimization of wall-climbing robot impeller by genetic algorithm based on computational fluid dynamics and kriging model. *Scientific Reports* **12**, 9571 (2022). https://doi.org:10.1038/s41598-022-13784-z

11 Ecovacs. *Ecovacs Window Cleaning Robot Manual*, <https://site-static.ecovacs.com/upload/uk/file/support/2023/11/07/031356_9841-WINBOTWIPROManual.pdf> (2023).

12 Staff, G. E. *Sandpaper Grit Charts & Grades*, <https://www.grainger.com/know-how/equipment/kh-video-sandpaper-grit-chart> (2023).

13 Wagner, M., Chen, X., Nayyerloo, M., Wang, W. & Chase, J. G. in *2008 IEEE/ASME International Conference on Mechtronic and Embedded Systems and Applications.* 210-215.
