# Supplementary material for: Snail-inspired water-enhanced soft sliding suction for climbing robots
Source: Nat Commun. 2024 May 13;15:4038. doi: 10.1038/s41467-024-48293-2 (PMC11091155; doi:10.1038/s41467-024-48293-2)
Supplement: Supplementary file 3 — Description of Additional Supplementary Files [file 41467_2024_48293_MOESM3_ESM.pdf]

Description of Additional Supplementary Files

Supplementary Movie 1. Sliding suction mechanism. This movie shows the fundamental design of a sliding suction cup, and demonstrates that the proposed suction cup can easily slide in water environments while a regular hydrophobic suction cup cannot.

Supplementary Movie 2. Working principle of the SSR. This movie shows how the proposed SSR slides upside down on a PMMA ceiling, and how the water residue can rapidly disappear via evaporation.

Supplementary Movie 3. Characterization of the SSR. This movie shows how we characterized the rotation and translation movement of SSR on substrates with different tilted angles.

Supplementary Movie 4. Demonstrations of the SSR. This movie shows four practical applications of SSR.
